# Colon Cancer Sidedness, Presentation, and Survival at Different Stages

**DOI:** 10.1155/2019/4315032

**Published:** 2019-02-21

**Authors:** Mark B. Ulanja, Mohit Rishi, Bryce D. Beutler, Mokshya Sharma, Darryll R. Patterson, Nageshwara Gullapalli, Santhosh Ambika

**Affiliations:** ^1^University of Nevada, Reno School of Medicine, Department of Internal Medicine, 1155 Mill Street, W-11, Reno, NV 89502, USA; ^2^Renown Institute for Cancer, 1155 Mill Street, W-11, Reno, NV 89502, USA

## Abstract

**Background:**

Several prognostic factors have been used to guide therapy for colon cancer (CC). However, the relationship between CC laterality (sidedness) and prognosis remains under investigation.

**Objectives:**

To assess the effect of laterality on CC presentation and survival, using a Surveillance, Epidemiology, and End Results (SEER) population-based cohort.

**Methods:**

A retrospective cohort study using data from the SEER program (2007-2015).

**Results:**

Of the 163,980 patients with CC, 85,779 (52.3%) presented with right-sided CC (RCC) and 78,201 (47.7%) with left-sided CC (LCC). Stage distributions were as follows: stage I, 24.1%; stage II, 27.3%; stage III, 28.2%; and stage IV, 20.4%. In an adjusted modified Poisson regression approach for risk ratio (RR), patients with LCCs were more likely to be male (RR = 1.14; 95% CI 1.12-1.15, p<0.001). As compared to stage I, stage II cancers (RR = 0.88, 95% CI 0.87-0.90, p<0.001) were less likely to be LCC. Stage IV CC was slightly less likely to be left-sided (RR = 0.98, 95% CI 0.98, 0.96-1.00, p = 0.028). The median overall survival (OS) for RCC was 87 months. The median OS for LCC was not established, as more than half of the patients diagnosed with LCC were still living at the time of the analysis. In adjusted Cox proportional Hazard model, individuals with stage I, III, and IV LCCs had superior OS as compared to those with matched-stage RCC (adjusted HR = 0.87; 95% CI 0.85-0.88, p<0.001). However, OS was worse among those with stage II disease who presented with LCC (adjusted Hazard ratio [aHR] = 1.06; 95% CI 1.02-1.11, p = 0.004). CC-specific survival (CSS) was superior for LCC versus RCC for stages III and IV but worse for II.

**Conclusions:**

In this population-cohort study, LCC is associated with superior OS and CSS survival. The overall survival advantage was attributed to stage I, III, and IV disease. Individuals presenting with stage II disease exhibit superior survival if the CC is right-sided.

## 1. Introduction

Colon cancer (CC) is one of the most common malignancies in the United States and represents the second leading cause of death in the Western world [1]. A number of prognostic factors are used to guide therapy, but the value of CC laterality (sidedness) in prognosis remains controversial. The differences between the right and left colon have been hypothesized to be due to histologic, genetic, and immunologic features, all of which may confer prognostic value. Notably, the right and left colon are anatomically and embryologically different: the proximal colon is derived from the midgut and is perfused primarily by branches of the superior mesenteric artery, whereas the distal colon and rectum are derived from the hindgut and receive blood via the inferior mesenteric artery.

Several studies have explored the prognostic value of laterality with inconsistent results. Indeed, while some investigators have reported superior survival among individuals with right-sided colon cancer (RCC), others have found no difference in survival between left- and right-sided diseases [2–4]. One 2016 study demonstrated that RCC is associated with prolonged survival using propensity score matching [4]. However, a meta-analysis [5] of 15 studies performed that same year showed a significant survival benefit for left-sided colon cancer (LCC). Further subgroup analyses demonstrated significant prognostic differences in Western countries. The 2016 American Society of Oncology annual meeting and the 2016 European Society of Medical Oncology annual meeting described poor survival for patients with metastatic RCC [6], especially those with RAS wild-type tumors [7, 8].

These conflicting findings and previously published studies [2, 3, 9] have renewed our interest in investigating the effect of laterality on CC survival.

## 2. Methods

### 2.1. Study Design and Study Population

**Figure 1:**
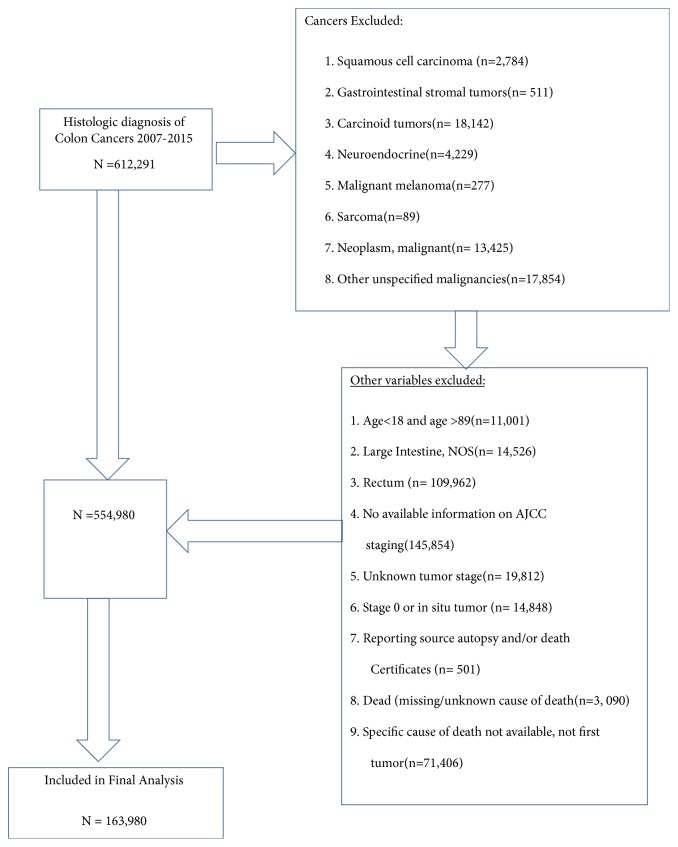
Patients' cohort flowchart showing exclusion and inclusion criteria.

This is a retrospective cohort study using the SEER database for identification of CC from all the registries captured in the SEER 18 program (San Francisco, Connecticut, Detroit, California, Kentucky, Louisiana, New Jersey, Greater Georgia, Hawaii, Iowa, New Mexico, Seattle, Utah, Alaska, San Jose-Monterey, Los Angeles, Rural Georgia, and Metropolitan Atlanta) who had a histologic diagnosis of colon cancers. SEER histology codes 8140, 8141, 8143, 8147, 8210, 8211, 8213, 8260, 8261, 8622, 8263, 8480, 8481, 8490, 8510, and 8560 were used for CC diagnosed between 2007 and 2015. The primary sites of tumor were determined using International Classification of Diseases for Oncology 3rd Edition (ICD-0-3), with the following site codes: C18.0, C18.2, C18.3, C18.4, C18.5, C18.6, C18.7, and C19-9. An index registry was used to classify patients into various geographic regions: Midwestern (Detroit and Iowa), Western (California, Los Angeles, San Francisco, Hawaii, New Mexico, Seattle, Utah, Alaska, and San Jose-Monterey), Southern (Rural Georgia, Kentucky, Louisiana, Metropolitan Atlanta, and Greater Georgia), and North Eastern (New Jersey and Connecticut). The SEER registries continuously code and submit American Joint Committee on Cancer (AJCC) 6th and 7th Edition stages for all cancers diagnosed in 2010 and beyond; patients diagnosed before 2010 are staged using the AJCC 6th edition only. The AJCC 6th edition was used in order to include all patients [10] diagnosed between 2007 and 2015. Exclusion criteria include (1) age younger than 18 years; (2) stage 0 or in situ tumor; (3) unknown tumor stage; (4) unknown site of primary tumor; (5) unavailable staging data; (6) patient deceased and cause of death unknown; and (7) history of previous cancer (Figure 1).

### 2.2. Data Source

The SEER database is comprised of data collected by the National Cancer Institute. The SEER program collects and publishes cancer incidence and survival data using population-based cancer registries that include approximately 28% of the population of the United States. The program routinely collects data on patient demographics, tumor sites, tumor morphology, staging, surgical treatment, and follow-up.

## 3. Main Outcome Measures

Our primary outcome of interest was overall survival (OS) and colon cancer-specific survival (CSS) between right and left-sided colon cancers. Secondary outcome was the likelihood of presentation as left or right-sided cancers, for stages I-IV. The right-sided cancers were calculated using cecum, ascending colon, hepatic flexure, and transverse colon, while left-sided cancers were calculated using splenic flexure, descending colon, sigmoid colon, and rectosigmoid junction. We estimated survival in months from the date of diagnosis to the date of death for nonsurvivors; the end of the follow-up period was used to ascertain survival for survivors. Patients were stratified into three groups based on age: young (<50 years [18-49 years]), middle-aged (50-69 years), and elderly (70 years or older [70-89]).

### 3.1. Statistical Analysis

The baseline characteristics and group differences were compared using Pearson's Chi square (X^2^) test for proportions. Nonparametric variables were compared using the Mann–Whitney-U test. The Kaplan-Meier method was used for survival analysis, and logrank test for equality of survival functions. Continuous variables were analyzed with the student t-test. Stepwise multivariable Cox regression models were built using the forward method, adjusting for baseline demographics, treatment, and tumor characteristics. Variables included in the adjusted models had a p-value <0.05 for the outcome of interest in the univariate analysis. These variables remained in the final model if they were still significant at P<0.05 in the final adjusted model, as a p-value <0.05 was deemed statistically significant in this study.

Modified Poisson approach with generalized linear model (glm) was used to estimate the risk ratio (RR) and confidence intervals calculated by using robust error variances method [11]. Model selection was done by using Akaike information criterion (AIC) [12]. Model with smallest AIC (244872) was selected. All statistical analyses were performed using Stata version 14.2 (StataCorp, College Station, Texas, USA).

## 4. Results

### 4.1. Study Population

Of the 612,291 patients with confirmed histologic diagnosis of CC, those with diagnosis as malignant neoplasm [n = 13,425], rectal cancer [n = 109,962], and other cancers of no interest were excluded (Figure 1). 163,980 patients were used in the final analysis. The exclusion and inclusion criteria for patients used in final analysis are shown in Figure 1.

### 4.2. Patient Characteristics

For the 163,980 patients [(80,181 (48.9%) female], 85,779 (52.3%) were right-sided CC (RCC) and 78,201 (47.7%) were left-sided CC (LCC). Mean ages (±SD) were [RCC (68.5 ± 12.7) and LCC (63.0 ± 13.0), p<0.001]. AJCC CC stage distributions were 24.1% stage I, 27.3% stage II, 28.2% stage III, and 20.4% stage IV (Table 1). For T4 colon cancers, right-sided cancers were more likely to be T4 (14,490 [54.6%]) versus left-sided colon cancers (12,069 [45.44]%), [p<0.001]. RCCs were also more likely to be N2 (14,311 [54.7%) versus LCC (11,865 [45.3%]), [p<0.001]. For stage IV disease, there was no difference in proportion between left-sided and right-sided cancers [p = 0.134].

In adjusted Modified Poisson regression approach for risk ratio (RR), patients with LCC were less likely to be middle age (50-69) (RR = 0.84; 95% CI 0.83-0.85, p<0.001), old (70-89) (RR = 0.61; 95% CI 0.60-0.62, p<0.001) as compared to young (<50 years). LCC individuals were also more likely to be male (RR = 1.14; 95% CI 1.12-1.15, p<0.001). Stage II cancers (RR = 0.88; 95% CI 0.87-0.90, p<0.001) were less likely to be LCC, and stage IV (RR = 0.98, 95% CI 0.96-1.00, P = 0.028) diseases only slightly, less likely to be LCC [Reference = Stage I]. Grades III (RR = 0.73; 95% CI 0.71-075) and IV (RR = 0.68; 95% CI 0.65-0.71) CCs were less likely to be LCC, [Reference = Grade I] (see Table 2).

### 4.3. Colon Cancer Laterality and Survival

**Figure 2:**
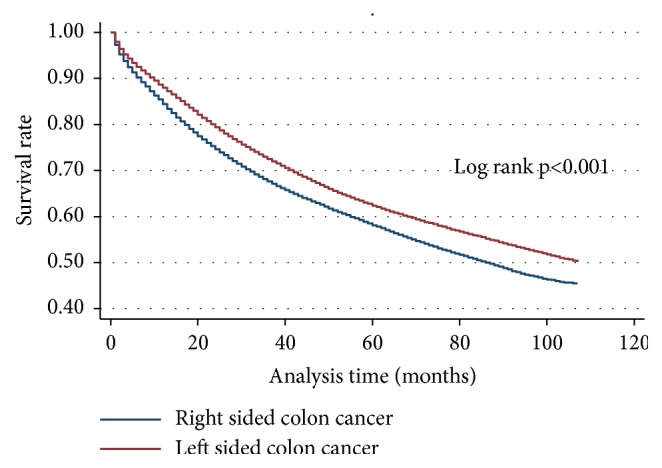
Kaplan-Meier survival function for overall survival (OS) for colon cancer laterality (sidedness). Right-sided colon cancer shows inferior OS over follow-up period. Left-sided colon cancer has superior OS survival over follow-up period.

**Figure 3:**
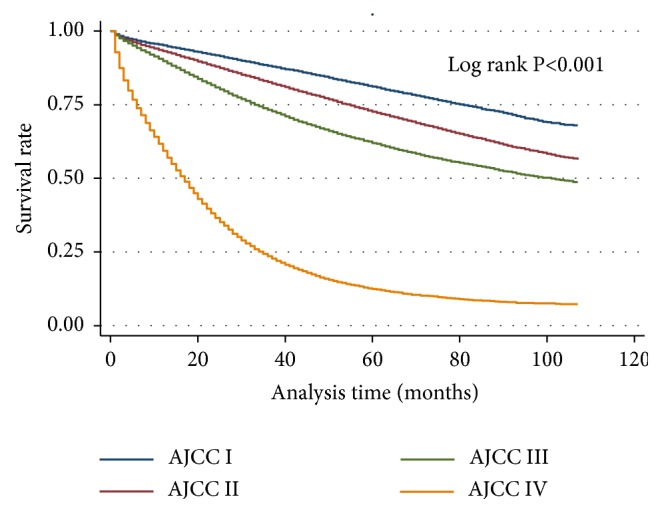
Kaplan-Meier survival function for overall survival (OS) for colon cancer stages. The AJCC I has superior OS, followed by AJCC II, and then AJCC III. The worst OS was in AJCC IV. AJCC I, AJCC II, AJCC III, and AJCC IV = American Joint Commission on Cancer (AJCC) stages 1, 2, 3, and 4, respectively.

**Figure 4:**
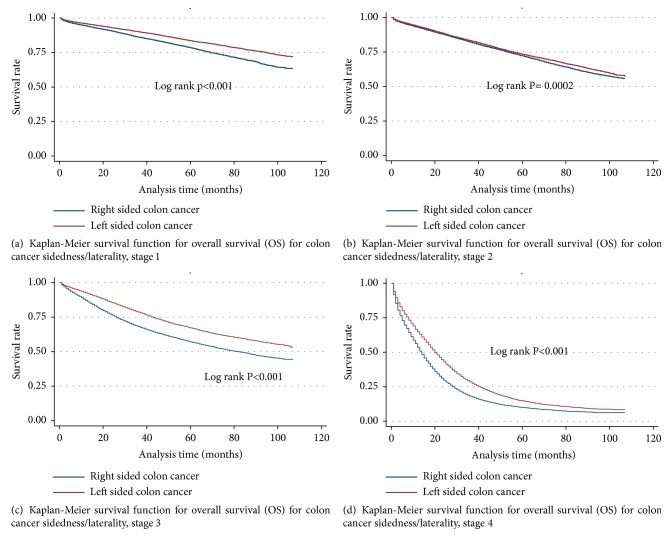


The median overall survival (OS) for right-sided colon cancer (RCC) was 87 months. The median OS for that of left-sided colon cancers (LCC) could not be determined, as greater than 50% of patients with LCC were still living at the time of the analysis (Figure 2). The median cancer-specific survival was not established for LCC or RCC, as more than half of the patients included in the dataset were still living at the time of the analysis (Supplementary Figure S1). Median OS for stages III and IV was 101 and 17 months, respectively (Figure 3), while median CSS for stages IV diseases was 18 months (Supplementary Figure S2). The OS for colon cancer stages stratified by sidedness is shown in Figures 4(a), 4(b), 4(c), and 4(d). In adjusted Cox proportional Hazard model, those with LCC had superior OS (adjusted HR = 0.87; 95% CI 0.85-0.88, p<0.001) [Table 3]. Stages I (aHR = 0.90; 95% CI 0.86-0.95, p<0.001), III (aHR = 0.85; 95% CI 0.82-0.88, p<0.001), and IV (aHR = 0.79; 95% CI 0.77-0.81, p<0.0001) had superior OS for LCC but worse OS for stage II (aHR = 1.06; 95% CI 1.02-1.11, p = 0.004) LCC.

The CC-specific survival (CSS) was better for LCC (aHR = 0.87; 95% CI 0.85-0.89, p<0.001) versus RCC. Although CSS was worse for LCC in stages II (aHR = 1.30, 95% CI 1.23-1.38, p<0.001), it was better for stages III (aHR = 0.84; 95% CI 0.80-0.87, p<0.001) and IV (aHR = 0.79; 95% CI 0.77-0.81, p<0.001) (Table 4).

For the entire cohort, the 3- and 5-year overall survival was 70.0% and 60.2%, respectively (p<0.05). The 3-year overall survival for RCC and LCC was 67.6% and 72.5%, respectively (p<0.001), while 5-year overall survival was 58.1% for RCC and 62.4% for LCC (P = 0.003).

## 5. Discussion

Our results demonstrate that laterality has an effect on OS and CSS for both early- and late-stage CC. LCC is associated with superior OS and CSS as compared to RCC among individuals presenting with stages I, III, or IV diseases. However, for reasons that remain to be elucidated, patients presenting with stage II disease exhibited inferior OS and CSS when the primary neoplasm was located on the left side. We also noted that individuals with LCCs were more likely to be young, whereas RCCs were more common among older cohort.

Our results are consistent with those reported in a 2017 study by Lim et al. [13]. Investigators performed a retrospective analysis of 414 South Korean patients and found that patients with RCC more frequently presented with larger neoplasms and more advanced nodal disease as compared to those with LCC. Individuals with RCC also exhibited inferior 5-year OS as compared to those with LCC (82.1% and 88.7%, respectively). Our analysis revealed similar findings: the 5-year OS of patients with RCC was significantly lower than that of patients with LCC at 58.1% and 62.4%, respectively.

A systematic review and meta-analysis by Petrelli et al. confirmed that LCC, as compared to RCC, is associated with a significantly reduced risk of death [14]. The Petrelli group analyzed over 1.4 million patients across 66 studies and concluded that “bearing a tumor on the left side of the colon [is] significantly associated with an absolute 19% reduced risk of death.” Notably, laterality was found to have a prognostic value that was independent of stage, race, and adjuvant chemotherapy. The Petrelli group also showed that the survival discrepancy between LCC and RCC is most significant among individuals with stage IV disease. Our analysis demonstrated that the overall survival advantage of LCC was primarily due to patients with stage I, III, and IV diseases. Indeed, left-sided tumors paradoxically represented a negative prognostic factor among patients with stage II disease (Table 4). The Petrelli group observed that the presence of microsatellite instability (MSI) was associated with a favorable outcome in stage II CC. Interestingly, for reasons that have yet to be established, stage II RCC is more likely than stage II LCC to be MSI-positive. Therefore, the prolonged survival associated with stage II RCC may be related to MSI.

It is important to acknowledge studies reporting contradictory results. In a recent population-based retrospective cohort study by Karim et al., authors used data from the province of Ontario, Canada, and found no significant difference in survival when comparing LCC and RCC and concluded that “disease laterality is not associated with long-term OS or CSS” [9]. Interestingly, however, investigators did observe that RCC was more likely to be staged as T4 and have poorly differentiated histologic features as compared to LCC; it is unclear why survival was similar between the two groups despite the more aggressive features associated with RCC. Limitations of the Karim group study include nonadjustments for confounders that represent prognostic factors in CC, such as race and ethnicity. Indeed, CC mortality rates vary significantly between different ethnic groups [15, 16], and thus not adjusting for these confounders was a significant limitation of the Karim et al.'s study.

The most significant decrease in survival associated with laterality is observed in patients with stage IV disease. Our results are consistent with other studies that demonstrate markedly decreased survival among individuals with RCC as compared to those with LCC [14, 17]. Indeed, this was conclusively demonstrated in two separate studies by Loupakis and Paski et al. [17, 18]. The Loupakis group evaluated the association between tumor location and survival parameters in patients with previously untreated stage IV CCs receiving first-line chemotherapy ± bevacizumab in three independent cohorts: a prospective pharmacogenetics study (PROVETTA) and two randomized phase III trials, AVF2107g and NO16966. In PROVETTA, patients with LCC exhibited superior OS. This was also the case in the AVF2107g and NO16966 trials. The authors concluded that primary tumor location is an important prognostic factor in previously untreated stage IV CC.

There are several hypotheses that may explain our findings. There are significant immunological differences between the proximal and distal colon [18]. Inflammation, epithelial injury, and increased cellular permeability are most common in the proximal region of the colon [19, 20]. These processes have been postulated to be due to interleukin-6 secreted by the unique microbiome present in that region of the bowel [21, 22]. It is therefore conceivable that the poor prognosis observed in RCC is due, in part, to a chronic inflammatory process with consequent carcinogenesis. Indeed, some authors have hypothesized that the downstream production of proinflammatory cytokines promotes aggressive CC through increased epithelial proliferation, impaired apoptosis, and/or angiogenesis [23, 24].

Microsatellite instability (MSI) colon cancers have a significantly better prognosis [25]. Right-sided colon cancers are known to have high MSI. The presence of MSI alone might not be able to explain the difference in mortality between right-sided and left-sided colon cancers. Phipps et al. [26] found increased MSI positivity in RCC, but the overall outcome and survival were still poor. Further work by Yamauchi et al. noted that frequencies of Cytosine-phosphate-Guanine (CpG) island methylator phenotype (CIMP-high), MSI-high, and BRAF mutations gradually increased from the rectum (<2.3%) to ascending colon (36-40%), followed by falls in the cecum (12-22%) [27]. The presence of BRAF mutations and CIMP-high mutations are associated with poorer prognosis [26, 27]. This may explain poorer overall survival for RCC in our cohort.

Interestingly, our analysis revealed that young individuals were more frequently affected by LCC whereas RCCs were more common among the elderly. The underlying cause of the relationship between age and tumor location has yet to be established. However, increasing age represents a negative prognostic factor in colon cancer [28]. Therefore, it is possible that the poorer overall survival observed in individuals with RCC in our cohort may be related to patient age, with accompanying multiple comorbidities.

Furthermore, the poorer OS and CSS associated with RCCs may be related to screening. Indeed, several studies have demonstrated that the lower incidence and mortality in LCCs are due to relatively early diagnosis using colonoscopy [29, 30]. While LCCs are more likely to present with obvious symptoms such as rectal bleeding and alteration in bowel habits leading to seeking early care, RCCs present more frequently with subtle symptoms such as microcytic anemia and weight loss which are not easily detectable until advanced stage [31, 32]. Our study shows that RCCs were more likely to be T4 and advanced nodal (N) stages, and this may be related to late diagnosis.

RCCs, as compared to LCCs, were significantly more likely to be mucinous (10.7% versus 5.0%) or signet cell ring carcinoma (1.4% versus 0.7%). This is consistent with previous reports in the literature [33, 34]. Mucinous adenocarcinoma produces mucin which dissects through tumor walls and promotes tumor extension; this portends a poor prognosis as well as a poor response to neoadjuvant and adjuvant chemotherapies [35, 36]. Indeed, the FIRE 3 [37] and CALGB/SWOG 80405 [8] trial subgroup analysis has shown that antiepidermal growth factor receptor therapy has a decreased benefit in patients with RCC. Signet ring carcinomas are aggressive and have a propensity for extensive intramural spread as well as peritoneal carcinomatosis [38]. Consequently, these tumors are associated with an overall poor prognosis [39]. The superior OS and CSS for LCCs may therefore be due to the lower propensity for mucinous and signet ring carcinomas to develop on the left side.

Our study has some limitations. First, because of its retrospective nature we could not assess causation. In addition, the study design is inherently prone to selection bias. Second, the SEER database does not include known prognostic factors such as smoking status, diet, and obesity nor does it include baseline data on comorbidities; this may therefore be subject to residual confounding, despite multivariable analysis. The SEER database also does not have information on nonsurgical cancer directed therapies. Furthermore, tumor markers such as MSI status and BRAF, which have prognostic value, could not be determined. Despite these limitations, the major strength of this study is the large sample size, which allows for a broad and generalizable perspective on presentation and survival for CC laterality.

## 6. Conclusions

In this population-cohort study, LCC have superior OS and CSS survival. The overall survival advantage was also noted for LCC in stages I, III, and IV; however worse survival was noted for stage II. LCC is independently less likely to present as stage II and IV diseases. The findings of this study may support laterality as a prognostic indicator in considering treatment for colon cancer.

## Figures and Tables

**Table 1:** Patient's demographic and clinical characteristics.

characteristics	Sidedness (laterality)		P-value
	Right sided cancer	Left sided cancer	
	N = 85,779 (%)	N = 78,201 (%)	
Age			
Mean (SD*∗*)	68.5 ± 12.7	63.0 ± 13.0	<0.001

Age group (years)			
*<50∗*	*6,798 (7.9)*	*11,546 (14.8)*	<0.001
50-69	35,523 (41.4)	41,258 (52.8)	
70-89	43,458 (50.7)	25,397 (32.5)	

Gender			
*Male *	*40,389 (47.1)*	*43,410 (55.5)*	<0.001
Female	45,390 (52.9)	34,791 (44.5)	

Race			
Hispanic	8,579 (10.0)	9,325 (11.9)	<0.001
Black	11,607 (13.5)	9,182 (11.7)	
White	59,370 (69.2)	50,391 (64.4)	
Others	6,223 (7.3)	9,303 (11.9)	

Insurance			
Insured	57,449 (67.0)	49,801 (63.7)	<0.001
Medicaid	9,362 (10.9)	10,553 (13.5)	
insured/no specifics	14,922 (17.4)	12,677 (16.2)	
uninsured	2,470 (2.9)	3,161 (4.0)	
unknown	1,576 (1.8)	2,009 (2.6)	

Marital status			
Married	44,909 (52.4)	42,824 (54.8)	<0.001
Divorced	7,971 (9.3)	7,483 (9.6)	
Separated	905 (1.1)	1,048 (1.3)	
Single	12,187 (14.2)	13,303 (17.0)	
Unknown	4,167 (4.9)	4,385 (5.6)	
Widowed	15,640 (18.2)	9,158 (11.7)	

Geographic region			
Northeastern	13,587 (15.8)	12,189 (15.6)	<0.001
Midwestern	8,801 (10.3)	6,789 (8.7)	
Western	41,869 (48.8)	40,144 (51.3)	
Southern	21,522 (25.1)	19,079 (24.4)	

*Tumor Grade*			
*Grade I*	*6,560 (7.7)*	*6,585 (8.4)*	
*Grade II*	*54,799 (63.4)*	*54,616 (69.8)*	
*Grade III*	*16,540 (19.3)*	*9,175 (11.7)*	*<0.001*
*Grade IV*	*2,713 (3.2)*	*1,292 (1.7)*	
*Unknown*	*5,167 (6.0)*	*6,533 (8.4)*	

*∗*AJCC (6th Edition stages)			
I	19,321 (22.5)	20,240 (25.9)	<0.001
II	25,989 (30.3)	18,744 (24.0)	
III	23,914 (27.9)	22,391 (28.6)	
IV	16,555 (19.3)	16,826 (21.5)	

Histology			
*Mucinous adenocarcinoma*	*9,172 (10.7)*	*3,923 (5.0)*	
*Adenocarcinoma NOS*	*59,460 (69.3)*	*57,095 (73.0)*	
*Signet ring cell carcinoma *	*1,219 (1.4)*	*523 (0.7)*	<0.001
*Adenocarcinoma in adenomatous polyps *	*5,849 (6.8)*	*8,117 (10.4)*	
*Others (papillary, adenosquamous, medullary)*	*10,079 (11.8)*	*8,543 (10.9)*	
Treatment by surgery			
Surgery performed	79,083 (92.2)	70,079 (89.6)	<0.001
No surgery (other reasons)	6,696 (7.8)	8,122 (10.4)	

T-staging			<0.001
T0	14(0.02)	44 (0.06)	
T1	11,707 (13.7)	16,304 (20.8)	
T2	11,871 (13.8)	9,311 (11.9)	
T3	44,314 (51.7)	36,608 (46.8)	
T4	14,490 (16.9)	12,069 (15.4)	
Tx	3,383 (3.9)	3,865 (4.9)	

N staging			
N0	49,302 (57.5)	44,227 (56.6)	<0.001
N1	20,546 (24.0)	20,155 (25.8)	
N2	14,311 (16.7)	11,865 (15.2)	
*∗*Nx	1,620 (1.9)	1,954 (2.5)	

*∗*SD = standard deviation, AJCC = American Joint Commission on Cancer, Nx = cancer in nearby lymph nodes cannot be measured, and <50 = 18-49.

**Table 2:** Independent predictors of association between left versus right sided (reference) colon cancers.

Characteristics	Adjusted Risk Ratio (RR) 95% Confidence Interval	P-value
*Male *	*1.14 (1.12-1.15)*	*<0.001*

Race		
Hispanic (*∗*ref)		
Black	0.84 (0.82-0.86)	<0.001
White	0.95 (0.94-0.97)	<0.001
Others	1.16 (1.14-1.18)	<0.001

Age		
<50 (ref)		
50-69	0.84 (0.83-0.85)	<0.001
70-89	0.61 (0.60-0.62)	<0.001

Insurance		
Insured (ref)		
Medicaid	1.08 (1.07-1.10)	<0.001
Insured/no specifics	1.02 (1.01-1.04)	0.002
Uninsured	1.05 (1.03-1.08)	<0.001
Unknown	1.10 (1.07-1.13)	<0.001

Marital status		
Married (ref)		
Divorced	1.01 (0.99-1.03)	0.345
Separated	1.04 (1.00-1.09)	0.042
Single	1.02 (1.01-1.04)	0.002
Unknown	1.03 (1.01-1.05)	0.007
Widowed	0.97 (0.95-0.99)	0.001

Geographic region		
Northeastern (ref)		
Midwestern	0.93 (0.91-0.95)	<0.001
Western	0.96 (0.95-0.98)	<0.001
Southern	0.97 (0.95-0.98)	<0.001

AJCC6th_edition		
Stage I (ref)		
II	0.88 (0.87-0.90)	<0.001
III	1.01 (0.99-1.02)	0.460
IV	0.98 (0.96-1.00)	0.028

*Tumor Grade *		
*Grade I (Ref)*		
*grade II*	*0.99 (0.97-1.01)*	*0.279*
*grade III *	*0.73 (0.71-0.75)*	*<0.001*
*grade IV*	*0.68 (0.65-0.71)*	*<0.001*
*Unknown*	*1.05 (1.02-1.07)*	*<0.001*

*Histology*		
*Mucinous adenocarcinoma (ref)*		
*Adenocarcinoma NOS∗*	*1.58 (1.54-1.62)*	*<0.001*
*Signet ring cell carcinoma*	*1.12 (1.04-1.21)*	*0.003*
*Adenocarcinoma in adenomatous polyps *	*1.75 (1.70-1.80)*	*<0.001*
*Others (papillary, adenosquamous, medullary)*	*1.42 (1.38-1.47)*	*<0.001*

Treatment		
No surgery (ref)		
Surgery performed	0.92 (0.90-0.94)	<0.001

*∗*ref = reference; NOS = not otherwise specified.

**Table 3:** Independent predictors of overall survival (OS) for colon cancer.

Characteristics	Adjusted HR (95% Confidence Interval)	P-value
Male	1.18 (1.16-0.1.20)	<0.001

Race		
Hispanic (ref)		
Black	1.15 (1.11-1.20)	<0.001
White	1.06 (1.02-1.09)	<0.001
Others	0.89 (0.85-0.92)	<0.001

Age		
*<50 (ref)*		
50-69	1.26 (1.22-1.30)	<0.001
70-89	2.60 (2.47-2.64)	<0.001

Insurance		
Insured (ref)		
Medicaid	1.39 (1.36-1.43)	<0.001
Insured/no specifics	1.14 (1.11-1.16)	<0.001
Uninsured	1.31 (1.25-1.37)	<0.001
Unknown	1.06 (1.00-1.13)	0.062

Marital status		
Married (ref)		
Divorced	1.21 (1.18-1.25)	<0.001
Separated	1.17 (1.08-1.26)	<0.001
Single	1.29 (1.26-1.32)	<0.001
Unknown	1.12 (1.07-1.17)	<0.001
Widowed	1.38 (1.35-1.42)	<0.001

Geographic region		
Northeastern (ref)		
Midwestern	1.08 (1.05-1.12)	<0.001
Western	1.06 (1.03-1.08)	<0.001
Southern	1.14 (1.11-1.17)	<0.001

AJCC6th edition		
Stage I (ref)		
II	1.30 (1.26-1.34)	<0.001
III	2.06 (2.00-2.12)	<0.001
IV	7.88 (7.63-8.13)	<0.001

*Tumor Grade *		
*Grade I (Ref)*		
*grade II*	*1.08 (1.04-1.12)*	*<0.001*
*grade III *	*1.47 (1.41-1.53)*	*<0.001*
*grade IV*	*1.58 (1.49-1.68)*	*<0.001*
*Unknown *	*1.18 (1.13-1.24)*	*<0.001*

*Histology*		
*Mucinous adenocarcinoma (ref)*		
*Adenocarcinoma NOS*	*0.90 (0.87-0.92)*	*<0.001*
*Signet ring cell carcinoma*	*1.37 (1.28-1.47)*	*<0.001*
*Adenocarcinoma in adenomatous polyps *	*0.74 (0.71-0.78)*	*<0.001*
*Others (papillary, adenosquamous, medullary)*	*0.82 (0.79-0.86)*	*<0.001*

Treatment		
No surgery (ref)		
Surgery performed	0.39 (0.38-0.40)	<0.001

Laterality		
Right sided (ref)		
Left sided	0.87 (0.85-0.88)	<0.001

*∗*ref = reference; *∗*HR = hazard ratio.

**Table 4:** Overall survival (OS) for colon cancer sidedness (laterality) for AJCC stages.

AJCC 6th edition stages	Unadjusted HR		Adjusted HR	
	(95% Confidence interval)		(95% Confidence interval)	
	Left vs Right	p-value	Left vs Right	P-value
I	0.72 (0.68-0.75)	<0.001	0.90 (0.86-0.95)	<0.001
II	0.93 (0.89-0.96)	<0.001	1.06 (1.02-1.11)	0.004
III	0.68 (0.66-0.71)	<0.001	0.85 (0.82-0.88)	<0.001
IV	0.74 (0.73-0.77)	<0.001	0.79 (0.77-0.81)	<0.001
All stages	0.76 (0.74-0.77)	<0.001	0.87 (0.85-0.88)	<0.001

## Data Availability

The data is available at https://seer.cancer.gov/data and can be accessed upon request.

## Supplementary Materials

Supplementary 1 Figure S1: Kaplan-Meier survival function for colon cancer-specific survival (CSS) for right-sided colon cancer (RCC) and left-sided colon cancer (LCC). The curve almost merges after 5 years of follow-up. The median colon cancer-specific survival could not be calculated from the curve as more than half of the patients diagnosed with colon cancer were still living at the time of the analysis.

Supplementary 2 Figure S2: Kaplan-Meier survival function for colon cancer-specific survival (CSS) for AJCC stages. The AJCC I has superior survival, followed by AJCC II and then AJCC III. Worst survival for AJCC IV. The median CSS could not be calculated for AJCC I-III, from the curve as more than half of the patients diagnosed with colon cancer were still living at the time of the analysis. AJCC I, AJCC II, AJCC III, and AJCC IV = American Joint Commission on Cancer (AJCC) stages 1, 2, 3, and 4, respectively.
