# Supplementary material for: Colon Cancer Sidedness, Presentation, and Survival at Different Stages
Source: J Oncol. 2019 Feb 21;2019:4315032. doi: 10.1155/2019/4315032 (PMC6409047; doi:10.1155/2019/4315032)
Supplement: Supplementary 1 — Figure S1: Kaplan-Meier survival function for colon cancer-specific survival (CSS) for right-sided colon cancer (RCC) and left-sided colon cancer (LCC). The curve almost merges after 5 years of follow-up. The median colon cancer-specific survival could not be calculated from the curve as more than half of the patients diagnosed with colon cancer were still living at the time of the analysis. [file 4315032.f1.docx]

Figure S1: Kaplan- Meier survival function for colon cancer specific survival (CSS) for right- sided colon cancer (RCC) and left-sided colon cancer (LCC). The curve almost merges after 5 years of follow up. The median colon cancer specific survival could not be calculated from the curve as more than half of the patients diagnosed with colon cancer were still living at the time of the analysis.
