# Supplementary material for: Colon Cancer Sidedness, Presentation, and Survival at Different Stages
Source: J Oncol. 2019 Feb 21;2019:4315032. doi: 10.1155/2019/4315032 (PMC6409047; doi:10.1155/2019/4315032)
Supplement: Supplementary 2 — Figure S2: Kaplan-Meier survival function for colon cancer-specific survival (CSS) for AJCC stages. The AJCC I has superior survival, followed by AJCC II and then AJCC III. Worst survival for AJCC IV. The median CSS could not be calculated for AJCC I-III, from the curve as more than half of the patients diagnosed with colon cancer were still living at the time of the analysis. AJCC I, AJCC II, AJCC III, and AJCC IV = American Joint Commission on Cancer (AJCC) stages 1, 2, 3, and 4, respectively. [file 4315032.f2.docx]

Figure S2: Kaplan- Meier survival function for colon cancer specific survival (CSS) for AJCC stages. The AJCC I has superior survival, followed by AJCC II, then AJCC III. Worst survival for AJCC IV. The median CSS could not be calculated for AJCC I-III, from the curve as more than half of the patients diagnosed with colon cancer were still living at the time of the analysis.

AJCC I, AJCC II, AJCC III, and AJCC IV= American Joint Commission on Cancer (AJCC) stages 1, 2, 3 and 4, respectively
